# Multidirectional activity of bakuchiol against cellular mechanisms of facial ageing ‐ Experimental evidence for a holistic treatment approach

**DOI:** 10.1111/ics.12784

**Published:** 2022-06-09

**Authors:** Anika Bluemke, Annika P. Ring, Jeannine Immeyer, Anke Hoff, Tanya Eisenberg, Wolfram Gerwat, Franziska Meyer, Sabrina Breitkreutz, Lina M. Klinger, Johanna M. Brandner, Grit Sandig, Marietta Seifert, Doerte Segger, Frank Rippke, Dorothea Schweiger

**Affiliations:** ^1^ Research and Development Beiersdorf AG Hamburg Germany; ^2^ Department of Dermatology and Venerology University Hospital Hamburg‐Eppendorf Hamburg Germany; ^3^ Gematria Test Lab GmbH Berlin Germany; ^4^ SGS Institute Fresenius GmbH Hamburg Germany

**Keywords:** antiageing, bakuchiol, claim substantiation in vivo/ex vivo/in vitro, retinol, skin physiology/structures

## Abstract

**Objective:**

Skin ageing is a multifactorial process involving formation of reactive oxygen species, consecutive inflammation with reduced epidermal and dermal cell viability and resulting damage to the extracellular matrix. Effective dermocosmetic treatment modalities should ideally address these hallmarks in a holistic approach. Here, we determined the corresponding activity profile of bakuchiol, a plant‐derived meroterpene, in an array of in vitro, ex vivo and in vivo studies and compared it to retinol, currently considered as gold standard in topical antiageing cosmetics.

**Methods:**

The antioxidative capacity and power of bakuchiol and retinol were analysed by measuring 2,2′‐diphenyl‐1‐picrylhydrazyl (DPPH) reduction via its absorption decay and electron spin resonance spectroscopy, respectively. Effects on prostaglandin E2 (PGE2), macrophage migration inhibitory factor (MIF), fibroblast growth factor 7 (FGF7), collagen type I and VII (COL1A1, COL7A1), fibronectin (FN) levels as well as the metabolization of water‐soluble tetrazolium 1 (WST‐1) were determined in human dermal fibroblasts. Epidermal regeneration was assessed utilizing an in vitro wound healing model. FN protein levels were analysed ex vivo after treatment with a formulation containing bakuchiol, retinol or vehicle using suction blister fluid. Skin condition improvement was determined in vivo in a split‐face comparison study after application of bakuchiol or vehicle.

**Results:**

In contrast to retinol, bakuchiol demonstrated high antioxidative efficacy. Levels of PGE2 and MIF were significantly decreased by both bakuchiol and retinol. Bakuchiol but not retinol significantly increased FGF7 protein levels. WST‐1 metabolization levels were significantly augmented by bakuchiol and retinol. Bakuchiol and retinol application led to a significant augmentation of COL1A1, COL7A1 and FN protein levels. Wounds supplemented with bakuchiol but not retinol displayed a significant increase in epidermis regeneration. Clinically, areas treated with a bakuchiol‐containing formulation showed a statistically significant increase in FN protein values after a 4‐week application compared to untreated areas and areas treated with vehicle.

**Conclusion:**

These data provide evidence for the multidirectional efficacy of bakuchiol against cellular hallmarks of skin ageing. Its activity profile shares some common features with retinol but demonstrates several hitherto unknown biopositive effects in our studies, namely stimulation of the critical extracellular matrix component FN, and accelerated epidermal regeneration and wound healing.

## Graphical abstract

In our study, we investigated the multidirectional effects of bakuchiol, a meroterpene isolated from the seeds of the plant *Psoralea corylifolia*, on cutaneous ageing. Bakuchiol exerted a broad range of antiageing effects as it acted (i) antioxidative (ii) anti‐inflammatory, increased (iii) cell activity, the expression of critical (iv) ECM components and improved (v) epidermal regeneration. These data demonstrate a multidirectional activity of bakuchiol against skin ageing and underline its potential as a holistic antiageing treatment approach.

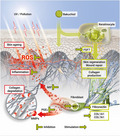

## Résumé

**Objectif:**

le vieillissement de la peau est un processus multifactoriel impliquant la formation de dérivés réactifs de l’oxygène, une inflammation consécutive qui entraîne une viabilité réduite des cellules du derme et de l’épiderme, et endommage la matrice extracellulaire. Pour être efficaces, les traitements dermocosmétiques devraient dans l’idéal traiter ces caractéristiques selon une approche holistique. Ici, nous avons déterminé le profil d’activité correspondant du bakuchiol, un méroterpène d’origine végétale, dans une série d’études in vitro, ex vivo et in vivo, et l’avons comparé au rétinol, qui est aujourd’hui considéré comme la référence parmi les cosmétiques anti‐âge topiques.

**Méthodes:**

la capacité antioxydante et la puissance du bakuchiol et du rétinol ont été analysées en mesurant la réduction du 2,2‐diphényl‐1‐picrylhydrazyl (DPPH) selon sa décroissance par absorption et à l’aide d’une spectroscopie par résonance magnétique électronique, respectivement. Les effets sur la prostaglandine E2 (PGE2), le facteur inhibiteur de la migration (FIM) des macrophages, le facteur de croissance des fibroblastes 7 (FGF7), le collagène de type I et VII (COL1A1, COL7A1), les taux de fibronectine (FN), ainsi que la métabolisation du tétrazolium 1 soluble dans l’eau (WST‐1) ont été déterminés dans des fibroblastes dermiques humains. La régénération épidermique a été évaluée à l’aide d’un modèle de cicatrisation des plaies in vitro. Les taux de fibronectine ont été analysés ex vivo après un traitement avec une formulation contenant du bakuchiol, du rétinol ou un excipient à l’aide d’un liquide d’aspiration sous forme de vésicules. L’amélioration de l’état de la peau a été déterminée in vivo dans une étude de comparaison en hémiface après l’application de bakuchiol ou d’un excipient.

**Résultats:**

Contrairement au rétinol, le bakuchiol s’est avéré présenter une efficacité antioxydante élevée. Les taux de PGE2 et de FIM ont significativement diminué avec le bakuchiol et le rétinol. L’application de bakuchiol s’est accompagnée d’une augmentation significative des taux de protéine FGF7, mais pas celle de rétinol. Les taux de métabolisation du WST‐1 ont augmenté de façon significative avec le bakuchiol et le rétinol. L’application de bakuchiol et de rétinol a entraîné une augmentation significative des taux de protéines COL1A1, COL7A1 et fibronectine. Les plaies supplémentées en bakuchiol, mais pas en rétinol, ont montré une augmentation significative de la régénération épidermique. Sur le plan clinique, les zones traitées avec une formulation contenant du bakuchiol ont montré une augmentation statistiquement significative des taux de fibronectine après une application de 4 semaines par rapport aux zones non traitées et aux zones traitées avec un excipient.

**Conclusion:**

ces données fournissent des preuves de l’efficacité multidirectionnelle du bakuchiol contre les caractéristiques cellulaires du vieillissement de la peau. Son profil d’activité partage certaines caractéristiques communes avec le rétinol, mais démontre plusieurs effets biopositifs jusqu’alors inconnus dans nos études : la stimulation de la fibronectine, composante essentielle de la matrice extracellulaire, et une régénération épidermique et une cicatrisation accélérée des plaies.

## INTRODUCTION

Aged skin is characterized by wrinkles, uneven pigmentation, skin roughness and laxity. These clinical signs are the result of structural and metabolic alterations caused by processes of intrinsic and extrinsic ageing. Intrinsic ageing has been ascribed to factors including telomere shortening, chronic inflammation, mitochondrial DNA single mutations and free radicals [1]. Aged skin further comprises a reduction of its antioxidative systems [2]. Also, the rate of cell proliferation declines due to the biological ageing process leading to a loss of skin structure and function. Extrinsic ageing is primarily triggered by UV irradiation and environmental influences. These insults result in skin damage that reinforces the chronological decline and accelerates cutaneous ageing. Human skin further loses the ability to cope with inflammatory conditions throughout ageing, resulting in a chronic proinflammatory state.

The topical application of retinoids such as retinoic acid, retinal or retinol is regarded as the clinical gold standard for an effective antiageing treatment [3, 4]. Molecular mechanisms of retinoids have been extensively described [5, 6, 7, 8, 9, 10]. Topical retinoids effectively reduce visible signs of ageing like wrinkles, laxity or roughness [4, 11] and decrease dyspigmentation of photodamaged skin including *livedo reticularis* and actinic lentigines [12]. However, topical treatment with retinoids can lead to concentration‐dependent skin dryness and irritation [13]. As the application of retinol causes minor adverse reactions compared to other retinoids such as retinoic acid [6, 14], it is a widely used active in the cosmetic treatment of facial ageing.

**FIGURE 1:**
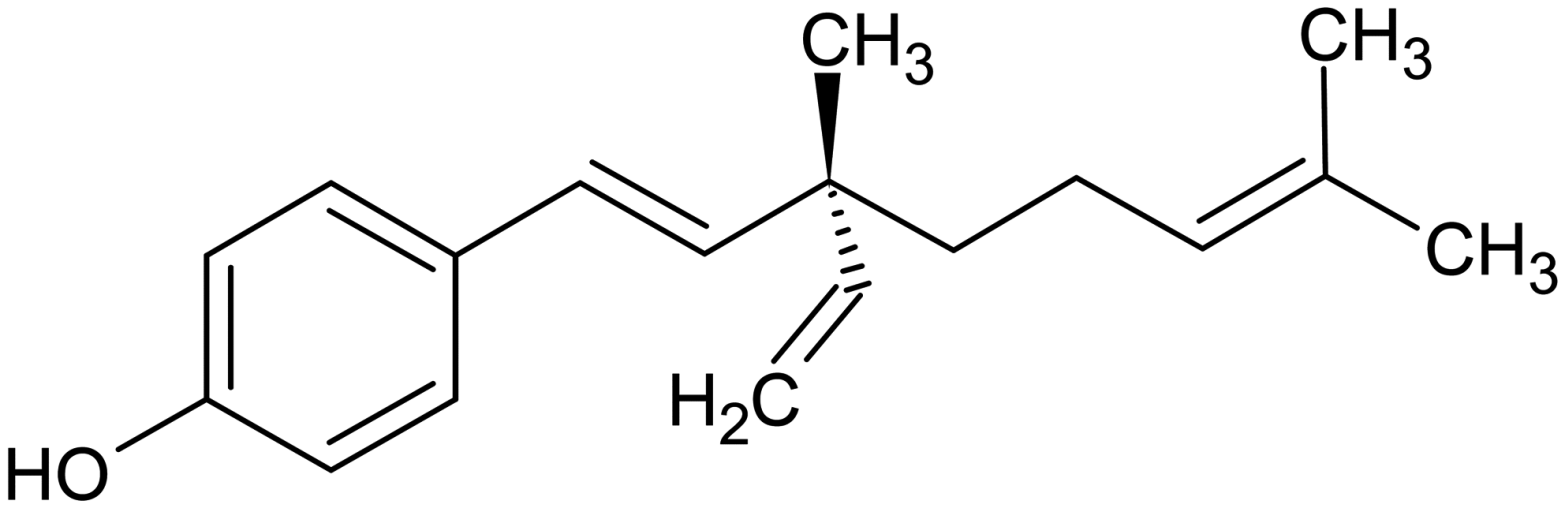
Structure of bakuchiol

In contrast to retinol that has been applied in skin care products since 1984 [15], bakuchiol has only recently gained attention as a topical antiageing compound. Bakuchiol is a meroterpene (Figure 1) that is derived from *Psoralea corylifolia* seeds. It has been used in traditional Indian and Chinese medicine for centuries [16, 17] and is well tolerated [18]. Bakuchiol was suggested to exhibit retinol‐like functions, as in a skin substitute model, both substances show similar gene expression patterns in vitro [19] and an improvement of cutaneous photodamage in vivo [20]. Hence, it has also been referred to as a plant‐derived functional retinoid analogue [21]. Further studies demonstrated antioxidant [19, 22, 23, 24], anti‐inflammatory [19, 25, 26, 27], antibacterial [28] as well as antiproliferative and antitumor effects [29, 30] of bakuchiol.

To effectively ameliorate and delay the multifactorial skin ageing processes, different cellular mechanisms need to be addressed in an integrated approach. Bakuchiol simultaneously modulates various targets rendering it a promising compound in this regard. As oxidative and inflammatory stresses are closely related to cutaneous ageing, their prevention by bakuchiol might promote the overall skin condition. However, the quality of the current scientific evidence has recently been critically appraised [31]. In this context, we determined the (i) antioxidative and (ii) anti‐inflammatory capacities of bakuchiol and retinol and examined their ability to improve the cellular metabolism and synthesis of growth factor 7 summarized as (iii) cell activity. We further analysed whether bakuchiol and retinol impact the expression of certain (iv) ECM components and improve (v) epidermal regeneration and re‐epithelization. Finally, an in vivo study was conducted to verify the clinical antiageing capacity of bakuchiol in human skin.

## MATERIALS AND METHODS

### Test materials

Bakuchiol was obtained from Sytheon Ltd (Boonton, New Jersey, United States). Retinol was purchased from Sigma‐Aldrich (St. Louis, Missouri, United States). For cell culture experiments, both test substances were freshly diluted in DMSO (Merck, Darmstadt, Germany). For the DMSO stock solutions, the applied concentration of test substances was 1000‐fold above the highest concentration applied in cell culture yielding 0.1% DMSO in media or buffer. Further dilutions were performed using media or buffer with 0.1% DMSO. Hence, all cell culture experiments were carried out in the presence of 0.1% DMSO. For the in vivo studies, topical retinol was formulated in the same vehicle as bakuchiol.

### In vitro studies

#### Determination of the antioxidative capacity

Bakuchiol, retinol (both applied at a final concentration of 100 μM) or the high standard trolox (Merck, final concentration of 25 μM) were diluted in DMSO. Briefly, 30 μL of these compounds (10‐fold higher concentrated than the final assay concentration) were added to a 96‐well flat bottom plate. Subsequently, 270 μL of 39 μg/mL DPPH (Merck, diluted in a 1:1 water/ethanol mixture) were quickly added to each well yielding a final concentration of 10% DMSO. As a control, the DPPH solution was incubated with DMSO lacking test substances. Further, blank controls were performed by incubating bakuchiol or retinol with water/ethanol (1:1) or only the water/ethanol (1:1) mixture containing 10% DMSO in the absence of DPPH. After 10, 30 and 60 min, the absorbance at 524 nm was measured using a Spark multimode microplate reader (Tecan, Männedorf, Switzerland). Signals of blank controls were subtracted.

#### Determination of the antioxidative power

Measurements of the antioxidative power (AP), which is a parameter to quantify both the antioxidant capacity and reactivity, were performed utilizing the X‐band electron spin resonance (ESR) spectrometer Miniscope MS 300 (Magnettech, Berlin, Germany). As described previously [32], DPPH (Sigma‐Aldrich) was used as a detector molecule. At least three concentrations of bakuchiol or retinol (diluted in 50% ethanol) were prepared and added to DPPH (dissolved in 96% ethanol) to obtain an initial radical concentration of 0.1 mM. The ESR signal intensity decay of each concentration of samples was recorded at different times during the reaction until saturation was reached and all antioxidant active molecules had reacted with the test radical.

The AP was calculated by means of the following equation:

$$\mathrm{AP}=\mathrm{RA}\times N_{\text{spins}}\;\left(\text{DPPH}\right)/t_{r}\times w_{c}$$

where RA is the constant reduction amplitude (1/e^2^), N_spins_ the quantity of reduced free radicals characterized by free electrons (spins) of DPPH, *t*_*r*_ the reduction time and *w*_*c*_ the characteristic weight of the antioxidant product (patent number: DE102005026133B4).

For a direct comparison of different antioxidants, the AP method is standardized to the activity of vitamin C (Sigma‐Aldrich). The antioxidative activity of a solution of 1 ppm vitamin C is defined as an antioxidative unit (AU).

#### Cell culture

Human dermal fibroblasts (HDFs) from multiple donors were obtained from Tissue Solutions Ltd (Glasgow, UK), Lonza (Basel, Switzerland), tebu‐bio (Heerhugowaard, Netherlands) or isolated from full‐thickness skin explants purchased from Alphenyx (Marseille, France) as described before [33]. Briefly, human full‐thickness skin explants were incubated for 2 h at 37°C in Dispase II solution (Roche, Penzberg, Germany) for separation of the dermis from the epidermis. To yield HDFs, de‐epidermized dermis explants were cultured in Dulbecco's Modified Eagle Medium (DMEM) containing 10% calf serum, 1% penicillin/streptomycin and 1% GlutaMAX (all from Thermo Fisher Scientific, Waltham, Massachusetts, United States). This medium was also applied for further culturing of HDFs. All suppliers from whom tissues or primary cells were purchased, performed tissue acquisition under regional authorization rules. Permission for the use of tissues in research applications was obtained by informed consent from the donor, nearest relative or by legal authorization.

#### Determination of PGE2 levels

HDFs were seeded in 96‐well plates with 10 000 cells/well in 100 μl medium containing 10% calf serum and incubated for 24 h. Subsequently, old medium was discarded, and medium containing 250 ng/mL lipopolysaccharides (LPS) isolated from *Salmonella minnesota* (Merck) was added to cells. Bakuchiol and retinol were applied in final concentrations of 1.25, 2.5, 5 and 10 μM. As controls, HDFs were treated with medium only or medium containing LPS. DMSO levels in the medium of controls were adjusted to 0.1%. As a further control, LPS and the high standard diclofenac (25 ng/mL, Merck) were jointly added to HDFs. Blank controls were performed by incubating medium without cells. After 24 h, supernatants were collected and frozen at −80°C. Supernatants were analysed using a commercially available PGE2 ELISA kit following the manufacturer's instructions (Cayman Chemical Company, Ann Arbor, Michigan, United States) and measured utilizing a SpectraMax microplate reader (Molecular Devices, San Jose, California, United States). Signals of blank controls were subtracted.

#### Determination of MIF protein levels

HDFs were seeded in 96‐well plates with 8000 cells/well in 100 μL medium containing 10% calf serum and incubated for 24 h. Depleted medium was discarded, and 100 μL medium containing bakuchiol or retinol in a final concentration of 1 μM or 10 μM was added. After 24 h of incubation, the medium was again removed, and cells were stressed by a 10 min incubation with 100 μl Dulbecco's phosphate‐buffered saline (DPBS) lacking calcium and magnesium (Thermo Fisher Scientific). As a control, cells were incubated with medium instead of DPBS. Subsequently, 100 μL fresh medium containing 1 μM and 10 μM bakuchiol or 1 μM and 10 μM retinol was added to the cells. After 24 h, supernatants were collected and frozen at −80°C. Cells treated with 0.1% DMSO lacking bakuchiol or retinol were used as an additional control. Blank controls were performed by incubating medium without cells. Supernatants were analysed using a commercially available MIF ELISA kit according to the manufacturer's instructions (Bio‐Techne GmbH, Wiesbaden, Germany). Measurements were performed using a Spark multimode microplate reader (Tecan). Signals of blank controls were subtracted.

#### Determination of FGF7 protein levels

HDFs were seeded in 6‐well plates with 150 000 cells/well in 2 mL medium containing 10% calf serum and incubated for 24 h. Subsequently, old medium was discarded, and cells were treated with medium containing 2% calf serum and bakuchiol or retinol in a final concentration of 10 μM. Control HDFs were supplemented with 0.1% DMSO lacking bakuchiol or retinol. Blank controls were performed by incubating medium without cells. After 24 h, the conditioned medium was transferred to Vivaspin protein concentrator spin columns (5000 MWCO; Sartorius, Göttingen, Germany) and concentrated approximately 10‐fold. Volumes of all medium supernatants were equalized by re‐addition of the flow through. FGF7 protein levels of the concentrated conditioned medium were analysed using a commercially available ELISA kit following the manufacturer's instructions (Bio‐Techne GmbH). Measurements were performed using a Spark multimode microplate reader (Tecan). Signals obtained from blank controls were subtracted and FGF7 protein levels were normalized to the total cell number that was determined utilizing a cell counter (Scepter, Merck).

#### Determination of WST‐1 metabolization

HDFs were seeded in 96‐well plates with 3000 cells/well in 100 μL medium containing 10% calf serum and incubated for 24 h. Depleted medium was discarded, and cells were treated with 100 μL medium containing retinol or bakuchiol in a final concentration of 1 μM or 10 μM, respectively. Control HDFs were supplemented with 0.1% DMSO lacking bakuchiol or retinol. As a further control, cells were treated with 10% triton‐X (Merck). Blank controls were performed by incubating medium without cells. After 72 h, cells were stained using the commercially available cell proliferation reagent WST‐1 according to the manufacturer's instructions (Merck). Absorbance was measured at 450 and 620 nm using a Tecan infinity M200 microplate reader (Tecan). The difference of these measurements was used for analysis. Signals of blank controls were subtracted.

#### Determination of COL1A1 and COL7A1 protein levels

HDFs were seeded in 96‐well plates with 10 000 cells/well in 100 μL medium containing 10% calf serum and incubated for 24 h. Bakuchiol or retinol were diluted in 100 μL medium without calf serum, and supplemented to the present medium in a final concentration of 1 μM or 10 μM, respectively. The control was supplemented with 100 μL serum‐free medium yielding a concentration of 0.1% DMSO corresponding to the retinol and bakuchiol treatment. As a high standard, 10 ng/mL transforming growth factor‐β (TGF‐β) and 11 μg/mL sodium ascorbate (both Merck) were applied. Blank controls were performed by incubating medium without cells. After 4 h, COL1A1 and COL7A1 protein levels of the conditioned medium were analysed using commercially available ELISA kits following the manufacturer's instructions (Novus Biologicals, Littleton, Colorado, United States). Measurements were performed using a Spark multimode microplate reader (Tecan). Signals of blank controls were subtracted. COL1A1 and COL7A1 protein levels were normalized to the total cell lysate protein amount that was determined using a commercially available bicinchoninic acid assay kit (Thermo Fisher Scientific) according to the manufacturer's instructions. A prerequisite for the use of the results was the positive reaction of cells to the high standard TGF‐β and sodium ascorbate.

For analysis of COL7A1 protein levels after an extended incubation time, HDFs were seeded in 96‐well plates with 3000 cells/well in 100 μL medium containing 10% calf serum. All treatments, controls and the analysis were performed as described above with the exception that bakuchiol and retinol were only used in a final concentration of 10 μM. Cells were harvested when subconfluence was reached, in particular, after 72 or 96 h.

#### Determination of FN protein levels

HDFs were seeded in 96‐well plates with 10 000 cells/well in 100 μL medium containing 10% calf serum and incubated for 24 h. Old medium was replaced by medium containing 2% calf serum and bakuchiol or retinol in a final concentration of 10 μM. The control was supplemented with 0.1% DMSO lacking bakuchiol or retinol. Blank controls were performed by incubating medium without cells. After 24 h, FN protein levels of the conditioned medium were analysed using a commercially available ELISA kit following the manufacturer's instructions (R&D Systems, Minneapolis, Minnesota, United States). Measurements were performed using a Spark multimode microplate reader (Tecan). Signals of blank controls were subtracted. FN protein levels were normalized to the total cell lysate protein amount that was determined as aforementioned.

#### Determination of epidermal regeneration in an in vitro wound healing model

The experiments were conducted as described previously [34]. For the treatment of wound healing models, bakuchiol or retinol were diluted in DPBS and added in a final concentration of 100 μM to the wound region (5 μL per wound). The reference wounds were supplemented with the same amount of DPBS containing 0.1% DMSO without bakuchiol or retinol. Additional wounds were left untreated as a further control. Wound healing models were incubated for 43 h at 95% humidity, 5% CO_2_ and 37°C. Subsequently, the samples were snap‐frozen in isopentane pre‐cooled with liquid nitrogen and stored at −80°C. Re‐epithelialization was evaluated in haematoxylin and eosin‐stained cryostat sections by measuring the length of the regenerated epidermis using a Leica DMLS microscope (10×), a Leica MC 170 HD CCD camera and the Leica LAS V4.9 software (Leica Microsystems, Wetzlar, Germany). Quantification was performed in a blinded fashion.

### In vivo studies I and II

For both in vivo studies, the recommendations of the current version of the Declaration of Helsinki and the guideline of the International Conference on Harmonization Good Clinical Practice were observed as applicable to a cosmetic study. The protocol of study I was approved by the Independent Ethics Committee Freiburg (feki code 08/2610). In both studies, all volunteers provided written, informed consent. Subjects had healthy skin, belonged to Fitzpatrick skin type I to III and the start, or change of hormonal medication was an exclusion criterion.

During a 10‐day preconditioning period and throughout the entire study period, subjects were required to refrain from UV exposure on the test areas. Sweat promoting activities were prohibited 24 h prior to scheduled assessments.

An investigator demonstrated the correct application of formulations using an amount, which corresponded to the usual skin care regimen of subjects.

### Study I: Ex vivo determination of FN protein levels

Out of 52 female subjects, who were enrolled into this vehicle‐controlled study, 33 subjects completed the study and data of 31 subjects (30–64 years, mean age: 50.9 years) were included in data analysis. Dropouts occurred due to the SARS‐CoV‐2 pandemic and personal reasons (13 subjects) as well as due to incompatibility reactions (five subjects, caused by retinol treatment). Retinol‐mediated incompatibility reactions as well as sampling issues caused different numbers of subjects tested for each condition (see results for details).

Seven days prior to scheduled assessments, the use of skin care products, cleansers and soaps on the forearms was prohibited. On the first study day, four test areas were established on the inner forearms. On two test areas, two verum formulations were applied containing 0.5% bakuchiol or 0.15% retinol, respectively. In an opinion published by the Scientific Committee on Consumer Safety in 2016, the applied retinol concentration was considered as a cosmetic treatment [35]. On the other two test areas, the corresponding vehicle was used, or the area was left untreated. The positioning of treatment locations was permuted. After 4 weeks of twice‐daily application of test formulations, volunteers returned to the test institute. In each test area three suction blisters (7 mm in diameter) were generated as previously described [36, 37]. Briefly, custom‐made suction blister cups were put onto the test areas and a vacuum of 550–850 mbar was applied. After approximately 90–150 min, when suction blisters had formed, the vacuum was released, and the fluid was aspirated from the blister using a 24‐gauge needle. Fluids were frozen immediately at −80°C on dry ice until analysis. FN protein levels were quantified in suction blister fluid samples using a commercially available ELISA kit (R&D Systems). Measurements were performed using a Spark multimode microplate reader (Tecan). FN levels were normalized to total protein levels of suction blister fluid samples that were determined as aforementioned.

### Study II: In vivo determination of skin condition improvement

In total, 43 female volunteers were enrolled into this vehicle‐controlled split‐face comparison study. According to medical assessments, subjects showed mixed skin types (dry, normal, oily and combination skin). 34 subjects (39–66 years, mean age: 56.2 years) completed the study and were included in the analysis. Dropouts were caused by technical problems and non‐compliance (eight subjects), as well as incompatibility reactions (one subject, caused by vehicle and bakuchiol treatment).

Two weeks prior to the beginning of the study and during the entire study period, volunteers were required to refrain from using self‐tanning products or intensive facial cosmetic treatments (e.g. removal of superficial skin layers). During the 10‐day preconditioning period and throughout the study, subjects were asked to abstain from performing permanent make‐up, eyelash and eyebrow treatments, eye masks and patches. Three days prior to the first assessment, subjects were asked to refrain from using face care products. On the evening prior to the scheduled grading, subjects were required to stop the application of decorative cosmetics.

In the first 7 days of the 10‐day preconditioning phase, subjects received a cream jar containing the study vehicle cream (lacking any information on the content) and applied it twice‐daily to their entire face. At baseline, volunteers performed a self‐grading. In particular, they visually assessed the overall appearance of their facial skin by observing its freshness and radiance as well as any signs of skin ageing. Thereby a visual analogue scale ranging from 1 (very fatigued, aged) to 10 (very fresh, no signs of aged skin) was applied. Then, subjects received two blinded cream jars containing the verum (vehicle containing 0.5% bakuchiol) or the vehicle, respectively, without any specification on the content. During the study period of 12 weeks, one facial side was treated twice‐daily with verum while the other facial side was treated twice‐daily with the vehicle. The allocation of treatments to the test sites was permuted. After 12 weeks of regular use, volunteers again performed the self‐grading as afore mentioned.

### Statistical analysis

Statistical analyses were performed using Microsoft Excel for Office 365 (Microsoft Corporation, Redmond, Washington, United States), SAS Software Package for Windows V9.4 (SAS Institute GmbH, Heidelberg, Germany) and GraphPad Prism V8 (GraphPad Software, San Diego, California, United States).

The normal distribution of data was assessed using a Shapiro–Wilk's test. If normal distribution was confirmed, a repeated measure analysis of variance (RM‐ANOVA) with post‐hoc pairwise comparison was performed. If the normality hypothesis was rejected, Blom‐transformed ranks of the original data were assessed using a RM‐ANOVA with post‐hoc pairwise comparison or original data were assessed by using a Wilcoxon sign rank test. All statistical tests were two‐sided at significance level alpha = 0.05.

## RESULTS

### In vitro studies

#### Determination of antioxidative effects

To analyse the (i) antioxidative effects of bakuchiol and retinol, we performed two different assays using DPPH as a detector molecule.

##### Antioxidative capacity

**FIGURE 2:**
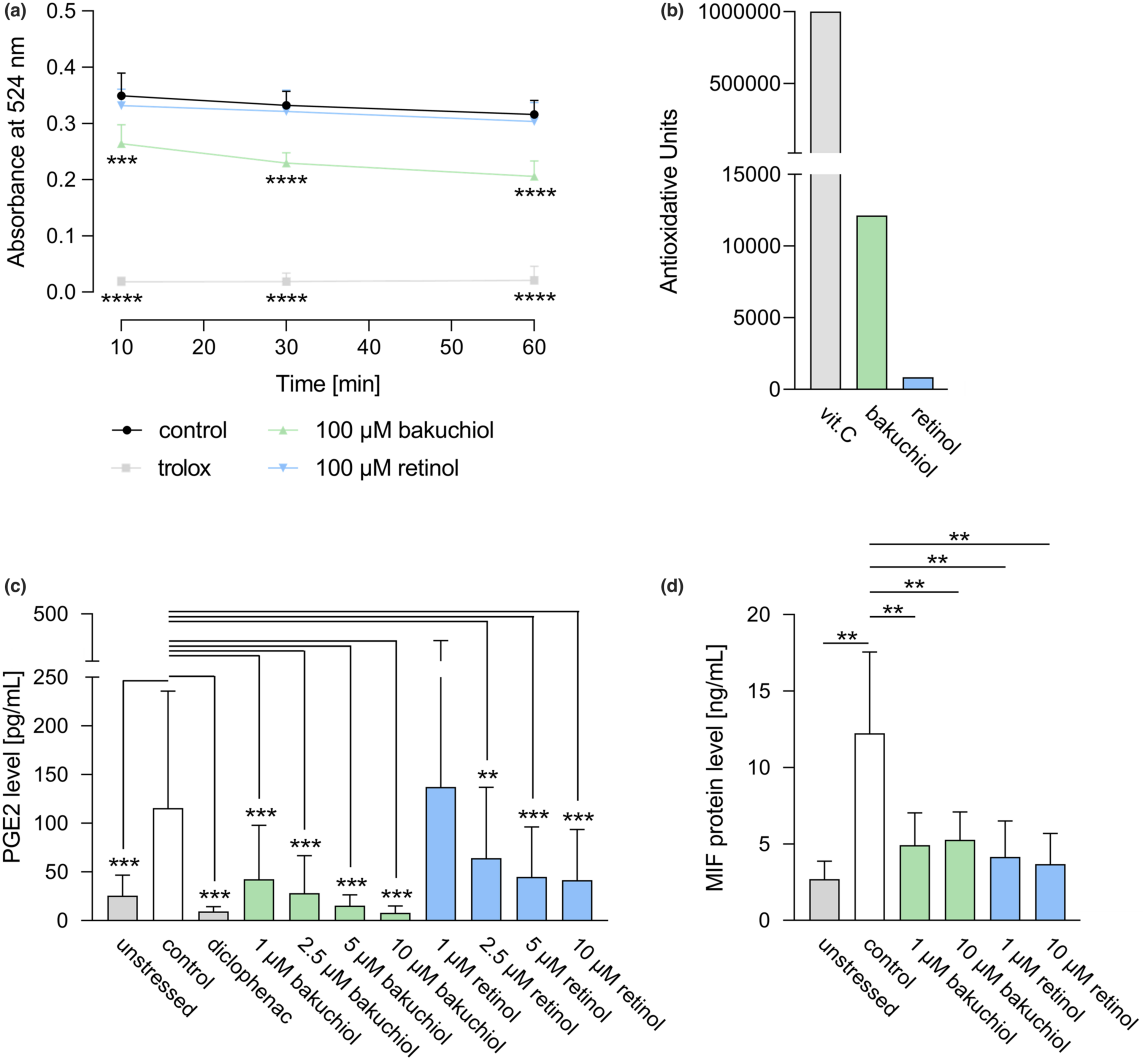
Antioxidative and anti‐inflammatory capacity of bakuchiol and retinol. (a) Antioxidative efficacy of bakuchiol (100 μM) or retinol (100 μM) compared to the high standard trolox (25 μM) and the solvent control determined by a DPPH antioxidant assay through absorbance measurement at 524 nm after 10, 30 and 60 min. *N* = 12. (b) Antioxidative power expressed in antioxidative units of bakuchiol, retinol or the high standard vitamin C (vit. C) determined using electron spin resonance spectroscopy. (c) ELISA‐based measurement of prostaglandin E2 (PGE2) levels in unstressed human dermal fibroblasts (HDFs), LPS‐stressed control HDFs, and in LPS‐stressed HDFs treated with the high standard diclofenac (25 ng/mL), bakuchiol or retinol (both applied at 1.25, 2.5, 5, 10 μM) for 24 h. *N* = 12. (d) Macrophage migration inhibitory factor (MIF) protein levels in unstressed HDFs, in HDFs stressed by a DPBS incubation and in stressed HDFs treated with bakuchiol or retinol (both applied at 1 and 10 μM) for 48 h determined by ELISA. *N* = 10. Results are depicted as mean ± SD. Statistics were performed by RM‐ANOVA with post‐hoc pairwise comparison based on Blom‐transformed ranks for Figure 2a or by a pairwise Wilcoxon signed rank test for Figure 2c and d. Significant differences are marked with an asterisk (**p ≤ 0.01, ***p ≤ 0.001, ****p ≤ 0.0001) [Colour figure can be viewed at wileyonlinelibrary.com]

The antioxidative capacity was determined by measuring the reduction of DPPH via its absorption decay. The high standard trolox showed a significantly elevated antioxidative capacity relative to the control (*p* = 0.0000 for all indicated time points) verifying proper measurement (Figure 2a). In relation to the control, the absorbance in bakuchiol‐treated samples was also significantly reduced at all timepoints investigated (10 min: *p* = 0.0003, 30 and 60 min: *p* = 0.0000) demonstrating an increased antioxidative capacity. In contrast, Retinol showed no significant antioxidative capacity compared to the control.

##### Antioxidative power

Electron spin resonance was applied to assay the AP of test substances, whereby a solution of 1 ppm vitamin C is defined as 1 AU. Retinol had a longer reaction time (2.59 min) than bakuchiol (0.99 min) or vitamin C (0.24 min) indicating a lower reactivity of retinol with free radicals. Further, the *w*_*c*_ values showed that bakuchiol (0.028 mg) had an increased capacity to react with free radicals compared to retinol (0.151 mg). Both these characteristics lead to an AP value of 12 125 AU for bakuchiol and 848 AU for retinol (Figure 2b).

#### Determination of anti‐inflammatory effects

For the investigation of the (ii) anti‐inflammatory effects of bakuchiol and retinol, we determined the level of the proinflammatory cytokines PGE2 and MIF.

##### PGE2 levels

Prostaglandin E2 levels of LPS‐treated HDFs were significantly elevated relative to the untreated control (*p* = 0.0005) indicating successful stress induction (Figure 2c). As expected, the pharmacologically well‐known high standard diclofenac induced significantly decreased PGE2 levels in LPS‐treated HDFs compared to the stressed control (*p* = 0.0005). In LPS‐ and bakuchiol‐treated cells, PGE2 levels were significantly reduced relative to HDFs only treated with LPS (*p* = 0.0005 for all indicated concentrations). Application of retinol at concentrations equal or higher to 2.5 μM also significantly diminished PGE2 levels in relation to the stressed control (2.5 μM: *p* = 0.0024; 5 and 10 μM: *p* = 0.0005).

##### MIF protein levels

As depicted in Figure 2d, stressed control HDFs displayed a significant increase in MIF protein levels relative to the unstressed control (*p* = 0.0020) demonstrating efficient stress induction. Treatment of stressed HDFs with bakuchiol resulted in significantly decreased MIF protein levels (1 μM: *p* = 0.0020; 10 μM: *p* = 0.0039) compared to the stressed control. Application of retinol also significantly lowered MIF protein levels (1 and 10 μM: *p* = 0.0020).

#### Analysis of cell activity

To examine the impact of bakuchiol and retinol on (iii) cell activity, FGF7 protein levels and WST‐1 metabolization were measured.

##### Determination of FGF7 protein levels

**FIGURE 3:**
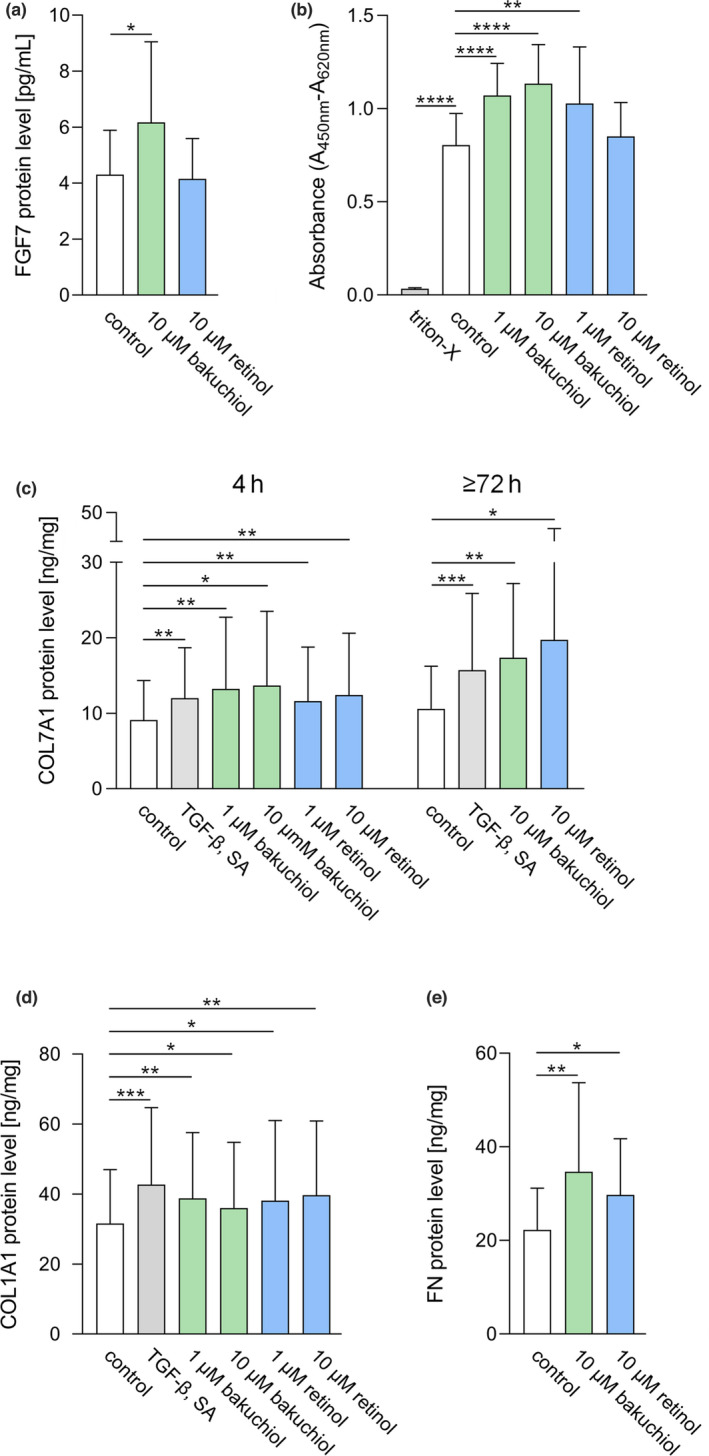
Effects of bakuchiol and retinol on cell activity and major ECM components. (a) Fibroblast growth factor 7 (FGF7) protein levels in control HDFs and in HDFs treated with 10 μM bakuchiol or retinol for 24 h. *N* = 13. (b) Quantification of WST‐1 metabolization in control HDFs and in HDFs treated with 10% triton‐X, bakuchiol or retinol (both applied at 1 and 10 μM) for 72 h. Difference in absorbance at 620 and 450 nm is depicted. *N* = 12. (c) Collagen, type VII, alpha 1 (COL7A1) protein levels in control HDFs and in HDFs treated with the high standard TGF‐β (10 ng/mL) and sodium ascorbate (SA; 11 μg/mL), as well as with bakuchiol or retinol (4 h: 1 and 10 μM, extended incubation: 10 μM for both test substances) for 4 h or for an extended incubation time (72 h or 96 h depending on cell confluence). *N* = 10 for 4 h, *n* = 11 for extended incubation. (d) Collagen, type I, alpha 1 (COL1A1) protein levels in control HDFs and in HDFs treated with the high standard TGF‐β and SA as aforementioned, bakuchiol or retinol (both applied at 1 and 10 μM) for 4 h. *N* = 11. (e) Fibronectin (FN) protein levels in control HDFs and in HDFs treated with 10 μM bakuchiol or retinol for 24 h. *N* = 11. All protein levels were determined by ELISA. Results are depicted as mean ± SD. Statistics were performed by RM‐ANOVA with post‐hoc pairwise comparison for Figure 3a, b and e or by a pairwise Wilcoxon signed rank test for Figure 3c and d. Significant differences are marked with an asterisk (*p ≤ 0.05, **p ≤ 0.01, ***p ≤ 0.001, ****p ≤ 0.0001) [Colour figure can be viewed at wileyonlinelibrary.com]

Treatment of HDFs with 10 μM bakuchiol significantly increased FGF7 protein levels relative to control cells (*p* = 0.0396), while 10 μM retinol displayed no significant effect (Figure 3a).

##### Determination of WST‐1 metabolization

As illustrated in Figure 3b, triton‐X significantly lowered WST‐1 metabolization levels in HDFs relative to control cells (*p* = 0.0000) indicating proper assay implementation. HDFs treated with 1 and 10 μM bakuchiol displayed significantly increased WST‐1 metabolization levels compared to the control (*p* = 0.0000 for both concentrations). Treatment with 1 μM retinol also caused a significant augmentation of WST‐1 metabolization levels (*p* = 0.0066) relative to control cells, while 10 μM retinol had no significant effect.

#### Expression of ECM components

To assess bakuchiol‐ and retinol‐mediated effects on the expression of (iv) ECM components, we determined COL7A1, COL1A1 and FN protein expression.

##### Determination of COL7A1 and COL1A1 protein levels

COL7A1 protein levels in cells treated with the high standard TGF‐β and sodium ascorbate were significantly higher relative to control cells (4 h: *p* = 0.0020, 72 or 96 h: *p* = 0.0010) as displayed in Figure 3c. Treatment of cells with bakuchiol or retinol in a concentration of 1 μM (*p* = 0.0020 for both test substances) and 10 μM (bakuchiol: *p* = 0.0195, retinol: *p* = 0.0059) significantly augmented COL7A1 protein levels already after 4 h compared to the control. After an extended incubation time, HDFs stimulated with 10 μM bakuchiol or retinol also displayed a significant increase in COL7A1 protein levels (bakuchiol: *p* = 0.0029, retinol: *p* = 0.0420) relative to control cells.

HDFs treated with the high standard TGF‐β and sodium ascorbate additionally showed a significant increase in COL1A1 protein levels (*p* = 0.0010) as shown in Figure 3d. Similarly, COL1A1 protein levels were significantly increased after 4 h of stimulation with bakuchiol (1 μM: *p* = 0.0020, 10 μM: *p* = 0.0322) or retinol (1 μM: *p* = 0.0244, 10 μM: *p* = 0.0098) relative to control cells.

##### Determination of FN protein levels

Figure 3e illustrates that HDFs treated with 10 μM bakuchiol or retinol demonstrated a significant increase in FN protein levels (bakuchiol: *p* = 0.0090, retinol: *p* = 0.0302) relative to control cells.

### Study I: Ex vivo determination of FN protein levels

**FIGURE 4:**
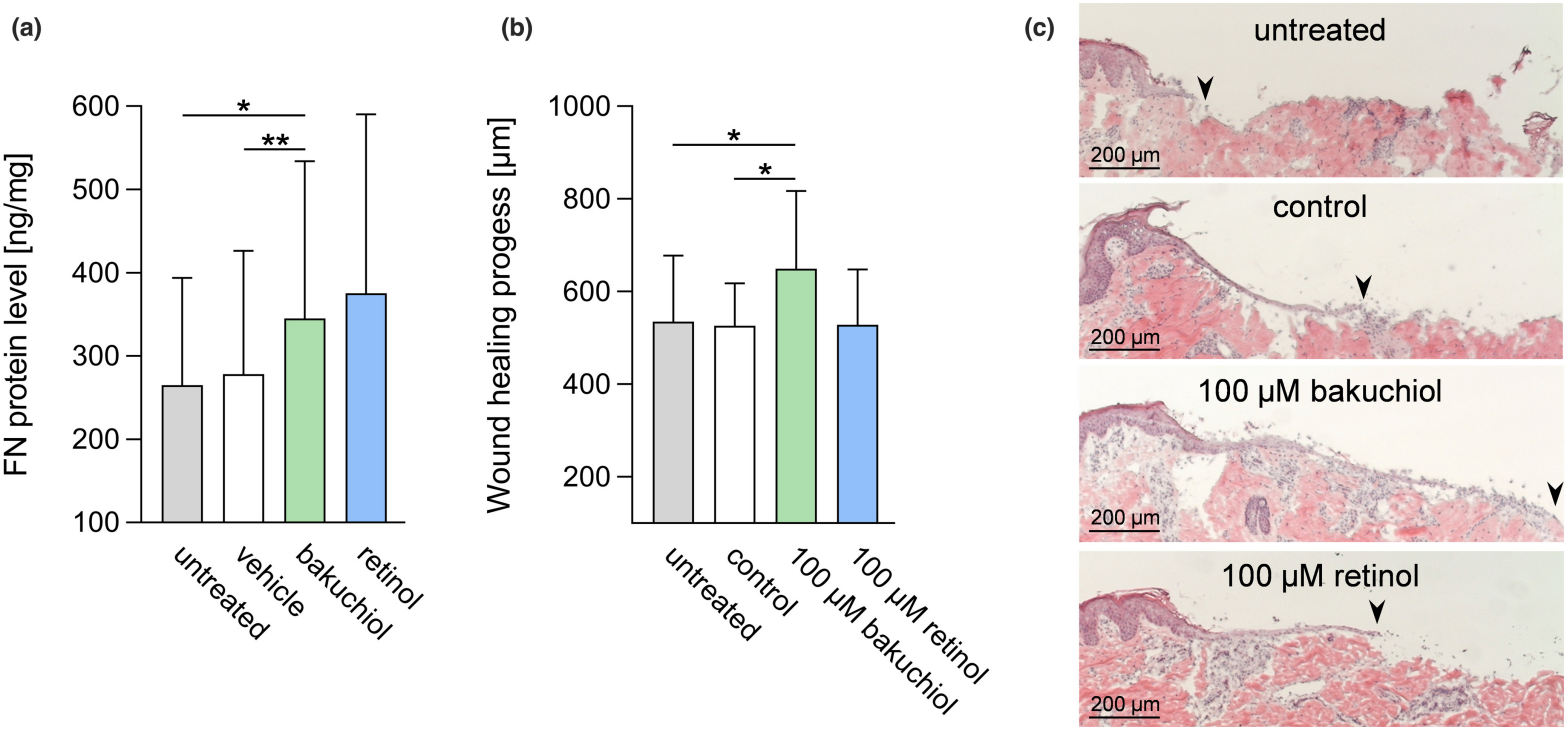
Analysis of bakuchiol and retinol in an ex vivo study and in an in vitro wound healing model. (a) ELISA‐based ex vivo determination of FN protein levels in suction blister fluids obtained from an untreated control site and after a 4‐week treatment (twice‐daily) with a formulation containing 0.5% bakuchiol, 0.15% retinol or vehicle. *N* = 26 (untreated), *n* = 29 (vehicle), *n* = 30 (bakuchiol), *n* = 19 (retinol). Epidermal regeneration in an in vitro wound healing model: (b) length of the regenerated epidermis 43 h after wounding in untreated and control wounds and in wounds treated with 100 μM bakuchiol or retinol. *N* = 11. (c) Examples for the progress of wound healing. Black arrows indicate the leading end of the regenerating epidermis. Results are depicted as mean ± SD. Statistics were performed by RM‐ANOVA with post‐hoc pairwise comparison. Significant differences are marked with an asterisk (*p ≤ 0.05, **p ≤ 0.01) [Colour figure can be viewed at wileyonlinelibrary.com]

An ex vivo study was carried out to investigate whether the previous data translate into ex vivo results. As depicted in Figure 4a, bakuchiol‐treated sites showed a statistically significant increase in FN protein levels in relation to untreated control areas (*p* = 0.0340) and areas treated with vehicle (*p* = 0.0088). Retinol‐treated sites displayed no significant alteration of FN protein levels in relation to untreated or vehicle‐treated areas. However, incompatibility reactions caused a lower number of subjects tested for retinol treatment (untreated: *n* = 26, vehicle: *n* = 29, bakuchiol: *n* = 30, retinol: *n* = 19). Additional minor deviations in the numbers of subjects tested were caused by sampling issues.

#### Improvement of the epidermal regeneration and re‐epithelization

To study the effects of bakuchiol and retinol on (v) epidermal regeneration and re‐epithelization, an in vitro wound healing model was applied. Figure 4b illustrates that bakuchiol‐treated wounds showed a significant increase in the length of the regenerated epidermis in relation to untreated (*p* = 0.0251) and control wounds (*p* = 0.0102). In contrast, wounds supplemented with retinol displayed no significant change in the length of the regenerated epidermis compared to both controls. Figure 4c exemplifies the progress of wound healing 43 h after bakuchiol or retinol treatment as well as in control and untreated wounds.

### Study II: In vivo determination of skin condition improvement

After 12 weeks of treatment with the bakuchiol‐containing formulation (t_1_), subjects (*n* = 34) rated the difference in the youthful appearance of their skin to the baseline determination (t_0_) with a mean t_1_‐t_0_ value of 2.57 ± 2.14. Compared to the t_1_‐t_0_ value of the vehicle‐treated site (2.06 ± 1.89), the youthfulness of the bakuchiol‐treated area was rated as significantly improved (*p* = 0.0275). Both treatments were rated as significantly better than baseline (*p* = 0.0000).

### In vivo studies: Tolerability

Results showed that the bakuchiol‐containing formulation in both in vivo studies was well tolerated. Over the entire duration of usage, one adverse skin reaction was observed, which was documented for both the bakuchiol‐containing formulation and the vehicle. After treatment with retinol‐containing formulations in study I, 23% of the entire panel of 52 subjects reported incompatibility reactions such as erythema, desquamation, dryness and itching, which led to the dropout of five subjects.

## DISCUSSION

Previous studies have implied that bakuchiol acts as a functional analogue of retinol [19, 20, 21]. Bakuchiol, thus, appears to be a promising alternative to retinol for facial antiageing treatments. As cellular ageing is multifactorial, we investigated effects of bakuchiol in comparison to retinol on different key processes to analyse its potential for a holistic treatment approach.

**FIGURE 5:**
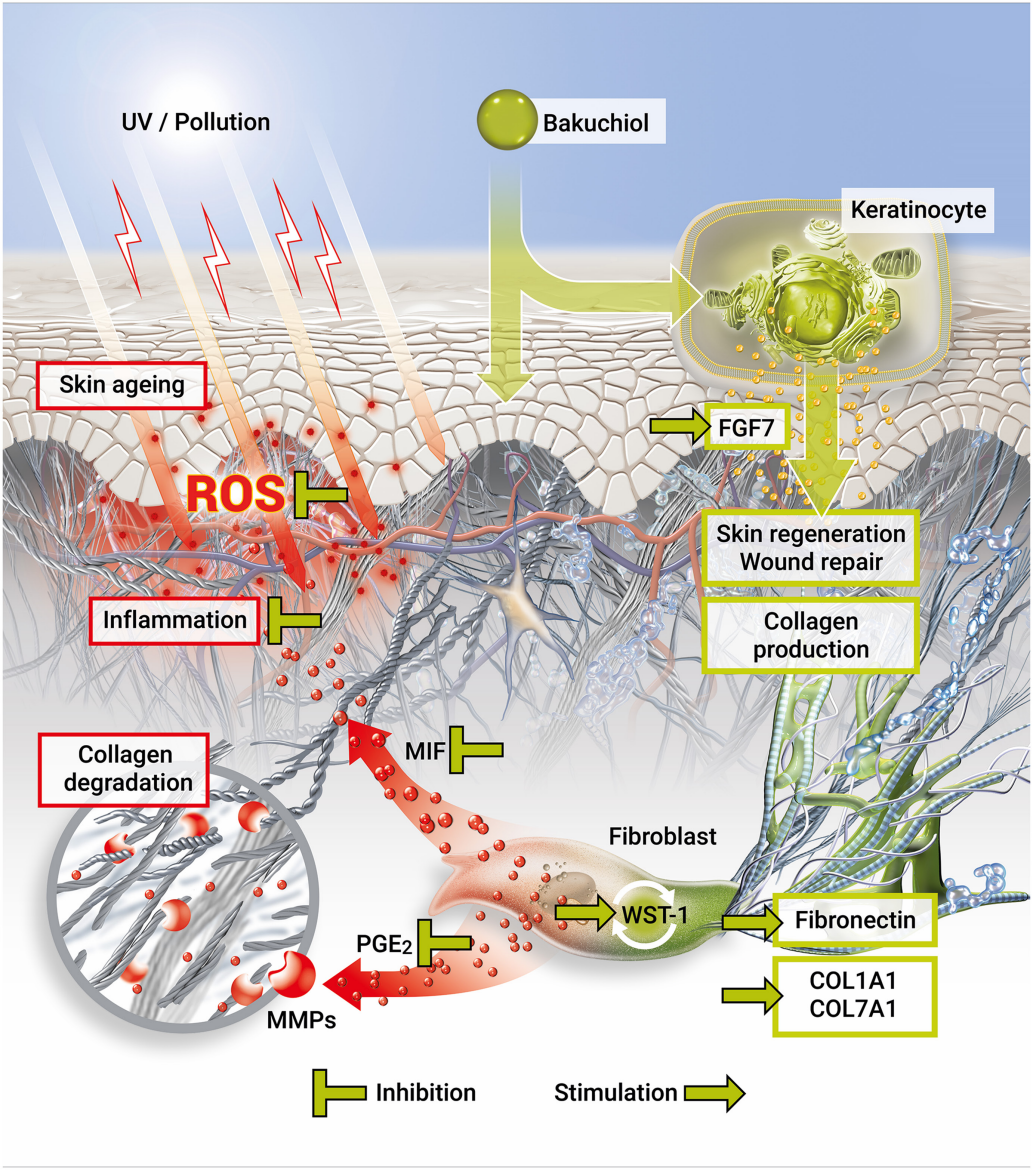
Schematic illustration of the multidirectional effects of bakuchiol counteracting skin ageing processes [Colour figure can be viewed at wileyonlinelibrary.com]

As depicted in (Table S1), we determined the (i) antioxidative and (ii) anti‐inflammatory capacities of bakuchiol and retinol. We further analysed how they influence (iii) cell activation, impact the formation of (iv) ECM components and (v) skin regeneration. In our investigation, we determined that bakuchiol shares functional similarities with retinol and at the same time exhibits unique, beneficial characteristics (Figure 5).

Our data demonstrated that bakuchiol but not retinol showed a high (i) antioxidative capacity and power. These data are in line with previous studies illustrating that bakuchiol decreases oxidative stress, prevents mitochondrial lipid peroxidation and protects mitochondrial function [22, 24, 38]. Retinol, however, has not been reported to exert antioxidative actions.

As the induction of ROS leads to inflammatory stress, we investigated the effects of bakuchiol and retinol on the expression of the two (ii) proinflammatory cytokines PGE2 and MIF.

We first analysed PGE2, which is a major prostaglandin generated in the human skin. PGE2 reduces collagen production and induces matrix metalloproteinase 1 (MMP‐1) expression in fibroblasts in vitro [39]. These PGE2‐mediated processes are cutaneous ageing mechanisms [39]. Thus, targeting PGE2 might be a promising strategy to oppose age‐associated collagen depletion [40]. Normally, low amounts of PGE2 are synthesized. However, in skin ageing, fibroblasts show elevated PGE2 levels [40, 41]. Herein, we show for the first time that bakuchiol and retinol significantly reduce PGE2 levels in HDFs in a dose‐dependent fashion. However, the effect induced by retinol was less pronounced than by bakuchiol. Our results are supported by a previous study using an in vivo inflammation model in which topically applied bakuchiol significantly reduced the PGE2 content in the arachidonic acid‐induced response [42]. Similarly, retinoids were shown to suppress PGE2 expression in human oral epithelial cells [43] and in human oral squamous carcinoma cells [44].

MIF is another proinflammatory cytokine that is ubiquitously expressed in various organs including the skin [45]. It is crucial for cell proliferation, angiogenesis and differentiation [46]. In the context of photoageing, both UVA and UVB irradiation increase MIF secretion by keratinocytes and dermal fibroblasts [46, 47]. Urschitz et al. reported a 4‐fold upregulation of MIF mRNA in photoaged preauricular skin [48]. Our results showed a significant, similar reduction of MIF protein levels in HDFs induced by bakuchiol and retinol indicating anti‐inflammatory properties. Indeed, it has been evidenced by earlier studies that bakuchiol exerts anti‐inflammatory actions [19, 25, 26, 27, 38]. However, the regulation of MIF protein levels by bakuchiol or retinol has not yet been documented.

Although PGE2 and MIF are both increased in cutaneous ageing [46, 49], their regulation occurs via two different signalling pathways. Therefore, the bakuchiol‐ and retinol‐induced decreases of both factors represent a broad anti‐inflammatory approach in antiageing treatment.

Oxidative and inflammatory stresses put the regenerative capacity of the skin at a serious risk. Further, cutaneous regeneration diminishes with age. We, therefore, investigated the effect of bakuchiol and retinol on the cutaneous regenerative capacity through analysing (iii) cell activity.

Keratinocytes are proposed to stimulate fibroblasts to synthesize growth factors, which, in turn, stimulate keratinocyte proliferation in a double paracrine manner [50]. The growth factor FGF7 is an example for such a mitogen [51]. It is also referred to as keratinocyte growth factor‐1 [51] and enhances the proliferation of keratinocytes [52] as well as their interaction with ECM components [53]. Our study demonstrates that bakuchiol‐treated HDFs showed significantly increased FGF7 protein levels. In contrast, FGF7 protein levels were slightly reduced by retinol treatment. This novel finding indicates that bakuchiol might support skin regeneration and repair processes by directly upregulating keratinocyte and indirectly increasing fibroblast proliferation. Bakuchiol thereby acts against the decline of growth factor levels that occurs during ageing [54].

Another factor that impacts the regenerative potential of the skin is the age‐associated reduction in the number [55] and growth rate [56] of dermal fibroblasts. Since an increase in WST‐1 metabolism indicates an improved cell viability [57], proliferation [58] and metabolic activity [59], we analysed WST‐1 metabolization after application of bakuchiol or retinol. Our results suggest that bakuchiol and to a certain extent also retinol can stimulate these cell activity‐related characteristics in HDFs.

In line with the reduction of cell activity, ageing skin is characterized by a diminished production of collagen and other ECM components as well as an augmented MMP expression [60, 61, 62, 63, 64, 65]. These alterations result in ECM damage, disturbed skin functions and subsequently the formation of wrinkles. We hypothesized that the increased fibroblast activity and decreased PGE2 and MIF levels mediated by bakuchiol could promote ECM components. Indeed, Chaudhuri and co‐workers showed that bakuchiol upregulates COL1A1 on gene and protein level [19]. To investigate the effects of bakuchiol and retinol on the ECM of HDFs, we analysed protein expression of the (iv) structural ECM factors COL1A1 and COL7A1 and the ECM adhesion factor FN.

COL1A1 is the most abundant structural protein in the skin [66]. However, aged fibroblasts display a reduced capacity for collagen synthesis [67]. COL7A1 forms anchoring fibrils in dermoepidermal junctions and enhances the mechanical skin stability [68]. During photoageing, COL7A1 levels decrease causing a weakened bond between the dermis and epidermis [69, 70, 71].

Our data demonstrate that bakuchiol and retinol increase COL1A1 levels confirming earlier observations. A previous study found out that bakuchiol significantly enhances expression levels of COL1 mRNA and significantly reduces MMP‐1 mRNA levels [72]. COL1A1 gene expression was shown to be augmented in vivo after 4 weeks of 0.1% retinol treatment [73]. Topical application of 0.4% retinol also significantly increased COL1A1 protein expression in the ECM in aged human skin in vivo [74]. However, our data clarify that in HDFs, COL1A1 and COL7A1 protein expression are increased already 4 h after stimulation with bakuchiol and retinol. We further show that COL7A1 protein expression persists at least for 72 h.

Another factor we investigated was the ubiquitous ECM adhesion protein FN found in two isoforms, namely plasma and cellular FN. It plays a crucial role in developmental processes, cell adhesion, migration and differentiation [75, 76]. Cellular FN is generated and assembled into fibril networks, impacting ECM homeostasis and ECM‐cell interactions [77]. Chronic UV exposure leads to a down‐regulation of FN gene expression in human skin biopsies [78]. Our data revealed a significant upregulation of cellular FN protein expression in HDFs after stimulation with bakuchiol and retinol. A previous in vivo study shows that topical treatment with 0.4% retinol leads to significantly increased FN protein levels in the ECM of aged human skin [74]. It has not yet been reported, though, that application of bakuchiol can induce enhanced FN protein expression in HDFs. To analyse whether these in vitro data translate into in vivo results, we determined the effect of bakuchiol and retinol on FN protein levels in an ex vivo study. After a 4‐week application, bakuchiol‐treated areas showed a significant increase in FN protein values compared to the vehicle. Retinol application also resulted in augmented FN protein levels; however, this effect was not significant. This might be caused by retinol‐mediated incompatibility reactions that reduced the number of subjects tested.

As a major component of the ECM, FN plays a crucial role in wound healing, being essential for tissue formation and connective tissue repair. FN functions in all phases of wound healing and thereby interacts with different cell types to build the ECM [79]. FGF7 is another important factor for wound healing. In acute human wounds FGF7 gene expression is rapidly up‐regulated. FGF7 mostly locates to dermal fibroblasts adjacent to the wound and in fibroblasts of the granulation tissue [52]. The wound healing process is delayed with ageing [80]. This is due to impaired cell proliferation and migration of fibroblasts and keratinocytes, a diminished reaction to growth factors and a decreased synthesis of ECM components [80]. These observations correlate with the general changes occurring during skin ageing [81]. Following aesthetic procedures such as Fraxel laser treatment the generation of micro‐wounds initiates microscopic wound healing processes leading to improved skin structure and rejuvenation [82]. Therefore, the ability of antiageing compounds to stimulate regenerative processes can indicate their skin rejuvenating potential. Considering the involvement of FN and FGF7 in wound healing and the bakuchiol‐induced upregulation of these factors in vitro, we next determined the effects of bakuchiol and retinol on (v) epithelial regeneration. Therefore, an in vitro wound healing model was applied [34]. The length of the regenerated epidermis of bakuchiol‐treated wounds was significantly increased, while retinol had no effect. These data reflect the more pronounced in vitro effect of bakuchiol on the wound healing‐associated parameters FGF7, FN and cellular metabolic activity when compared to retinol.

To determine whether bakuchiol, besides its biopositive activities, also improves the perceived skin appearance, a second self‐grading based in vivo study was performed. Study participants graded the youthfulness of their facial skin. When compared to baseline self‐grading at t_0_, treatment with both the vehicle and the bakuchiol‐containing formulation for 12 weeks significantly enhanced the perceived skin appearance. The vehicle was selected to be as little nourishing as possible. However, a certain improvement in self‐grading, especially regarding measurement at t_0_ after 3 days of not using any skin care products, cannot be excluded. Nonetheless, after application of the bakuchiol‐containing formulation, subjective grading of the youthful skin appearance was significantly increased compared to the corresponding vehicle with regard to t_1_‐t_0_ values.

In our in vivo studies, bakuchiol had a good skin compatibility. This is in line with a previous study showing that a bakuchiol‐containing moisturizer was well tolerated in subjects with sensitive skin [18]. In contrast, retinol application performed in study I caused skin irritations in several volunteers. It is well documented that retinol can induce various skin issues including erythema, itching, desquamation or papules [6, 14]. Further, retinoids are associated with photosensitization and are degraded by exposure to air or light to biologically inactive substances [11]. Hence, the efficacy of retinol in an antiageing treatment strongly depends on its delivery mode. Bakuchiol, on the other hand, is photostable and can be applied diurnally. The photostabilizing effect of bakuchiol on retinol, as demonstrated by Chaudhuri et al. [83], provides a promising rationale for the combination of both compounds.

Our results expand the scientific knowledge about bakuchiol and advance our understanding of cutaneous effects exerted by retinol. Figure 5 summarizes the proposed actions of bakuchiol. Moreover, our data provide evidence for the multidirectional efficacy of bakuchiol against several cellular hallmarks of skin ageing, exceeding the effects of plant‐derived functional retinoid analogues.

## CONCLUSION

Treatment with bakuchiol provides an advanced, holistic and multidirectional treatment approach for skin ageing as it acts (i) antioxidative, (ii) anti‐inflammatory, impacts (iii) cell activity, increases the expression of critical (iv) ECM components and improves (v) epidermal regeneration and re‐epithelization.

## CONFLICT OF INTEREST

Anika Bluemke, Annika P. Ring, Jeannine Immeyer, Anke Hoff, Tanya Eisenberg, Wolfram Gerwat, Franziska Meyer, Sabrina Breitkreutz, Lina M. Klinger, Frank Rippke and Dorothea Schweiger are employees of Beiersdorf AG. Grit Sandig and Marietta Seifert are employees of the Gematria Test Lab GmbH. Doerte Segger is an employee of the SGS Institute Fresenius GmbH. None of the authors state a conflict of interest.

## Supporting information


Table S1
