# Supplementary material for: Multidirectional activity of bakuchiol against cellular mechanisms of facial ageing ‐ Experimental evidence for a holistic treatment approach
Source: Int J Cosmet Sci. 2022 Jun 9;44(3):377–93. doi: 10.1111/ics.12784 (PMC9328396; doi:10.1111/ics.12784)
Supplement: Supplementary file 1 — Table S1 [file ICS-44-377-s001.docx]

**Table 1: Multifactorial activities of bakuchiol and retinol against cellular aging mechanisms determined by *in vitro*, *ex vivo* and *in vivo* studies**

| **Activity** | **Aging parameter** | **Concentration** | | **Significant effects** | | **Results (mean)** | | | | **Test system** |
| --- | --- | --- | --- | --- | --- | --- | --- | --- | --- | --- |
|  |  | **bakuchiol** | **retinol** | **bakuchiol** | **retinol** | **bakuchiol** | **retinol** | **high standard** | **control** |  |
| (i)  Antioxidative capacity and power | Antioxidative capacity | 100 µM | 100 µM | 10 min: + | 10 min: 0 | 0.26 (A_524_) | 0.33 (A_524_) | 0.02 (A_524_) | 0.35 (A_524_) | Cell free  assay (DPPH) |
|  |  |  |  | 30 min: + | 30 min: 0 | 0.23 (A_524_) | 0.32 (A_524_) | 0.02 (A_524_) | 0.33 (A_524_) |  |
|  |  |  |  | 60 min: + | 60 min: 0 | 0.21 (A_524_) | 0.30 (A_524_) | 0.02 (A_524_) | 0.32 (A_524_) |  |
|  | Antioxidative power | n/a | n/a | high | low | 12125 AU | 848 AU | 1000000 AU | n/a | ESR  (DPPH) |
| (ii)  Anti-  inflammatory effects | PGE2 | 1.25 µM | 1.25 µM | + | 0 | 42.5 pg/mL | 137.3 pg/mL | 9.3 pg/mL | unstressed:  25.6 pg/mL  stressed:  115.7 pg/mL | Human dermal primary fibroblasts |
|  |  | 2.5 µM | 2.5 µM | + | + | 28.2 pg/mL | 64.1 pg/mL |  |  |  |
|  |  | 5 µM | 5 µM | + | + | 15.4 pg/mL | 44.9 pg/mL |  |  |  |
|  |  | 10 µM | 10 µM | + | + | 8.0 pg/mL | 41.6 pg/mL |  |  |  |
|  | MIF | 1 µM | 1 µM | + | + | 4.9 ng/mL | 4.2 ng/mL | n/a | unstressed:  2.7 ng/mL  stressed:  12.2 ng/mL |  |
|  |  | 10 µM | 10 µM | + | + | 5.3 ng/mL | 3.7 ng/mL |  |  |  |
| (iii)  Cell activation | WST-1 | 1 µM | 1 µM | + | + | 1.07 (A_450_-A_620_) | 1.03 (A_450_-A_620_) | 0.03 (A_450_-A_620_) | 0.80 (A_450_-A_620_) |  |
|  |  | 10 µM | 10 µM | + | 0 | 1.13 (A_450_-A_620_) | 0.85 (A_450_-A_620_) |  |  |  |
|  | FGF7 | 10 µM | 10 µM | + | 0 | 6.2 pg/mL | 4.2 pg/mL | n/a | 4.3 pg/mL |  |
| (iv) ECM modification (*in vitro*) | COL7A1  (4h) | 1 µM | 1 µM | + | + | 13.2 ng/mg | 11.6 ng/mg | 12.0 ng/mg | 9.1 ng/mg |  |
|  |  | 10 µM | 10 µM | + | + | 13.7 ng/mg | 12.4 ng/mg |  |  |  |
|  | COL7A1  (72 h or 96 h) | 10 µM | 10 µM | + | + | 17.3 ng/mg | 19.7 ng/mg | 15.7 ng/mg | 10.6 ng/mg |  |
|  | COL1A1 (4h) | 1 µM | 1 µM | + | + | 38.8 ng/mg | 38.1 ng/mg | 42.7 ng/mg | 31.6 ng/mg |  |
|  |  | 10 µM | 10 µM | + | + | 36.0 ng/mg | 39.7 ng/mg |  |  |  |
|  | FN | 10 µM | 10 µM | + | + | 34.7 ng/mg | 29.7 ng/mg | n/a | 22.2 ng/mg |  |
| (iv) ECM modification (*ex vivo*) | FN | 0.5% | 0.15% | +  (vs. vehicle, untreated) | 0 | 357.1 ng/mg | 360.3 ng/mg | n/a | untreated:  262.9 ng/mg  vehicle:  278.1 ng/mg | *Ex vivo*  (suction blister fluid) |
| (v) Epidermal regeneration and wound healing | Re-epitheliali-zation | 100 µM | 100 µM | +  (vs. control, untreated) | 0 | 649 µm | 529 µm | n/a | untreated:  535 µm  control:  526 µm | *In vitro* wound healing model |
| Youthful facial appearance | Self-assessment | 0.5% | not tested | +  (vs. vehicle, baseline) | not tested | t_1_-t_0_: 2.57 | not tested | n/a | t_1_-t_0_: 2.06 | *In vivo* (split face design) |
